# Patients with chronic kidney disease are at an elevated risk of dementia: A population-based cohort study in Taiwan

**DOI:** 10.1186/1471-2369-13-129

**Published:** 2012-09-29

**Authors:** Kao-Chi Cheng, Yu-Lung Chen, Shih-Wei Lai, Chih-Hsin Mou, Pang-Yao Tsai, Fung-Chang Sung

**Affiliations:** 1 Department of Family Medicine, China Medical University Hospital, Taichung, 404, Taiwan; 2 School of Medicine, China Medical University, 91 Hsueh-Shih Road, Taichung, 404, Taiwan; 3 Management Office for Health Data, China Medical University Hospital, Taichung, 404, Taiwan; 4 Department of Public Health, China Medical University, Taichung, 404, Taiwan

**Keywords:** Chronic kidney disease, Comorbidity, Dementia, Medication, Retrospective cohort study

## Abstract

**Background:**

Chronic kidney disease (CKD) is more prevalent in Taiwan than in most countries. This population-based cohort study evaluated the dementia risk associated with CKD.

**Methods:**

Using claims data of 1,000,000 insured residents covered in the universal health insurance of Taiwan, we selected 37049 adults with CKD newly diagnosed from 2000–2006 as the CKD cohort. We also randomly selected 74098 persons free from CKD and other kidney diseases, frequency matched with age, sex and the date of CKD diagnosed. Incidence and hazard ratios (HRs) of dementia were evaluated by the end of 2009.

**Results:**

Subjects in the CKD cohort were more prevalent with comorbidities than those in the non-CKD cohort (*p* <0.0001). The dementia incidence was higher in the CKD cohort than in the non-CKD cohort (9.30 vs. 5.55 per 1,000 person-years), with an overall HR of 1.41 (95% confidence interval (CI), 1.32-1.50), controlling for sex, age, comorbidities and medicaitions. The risk was similar in men and women but increased sharply with age to an HR of 133 (95% CI, 68.9-256) for the elderly. However, the age-specific CKD cohort to non-CKD cohort incidence rate ratio decreased with age, with the highest ratio of 16.0 (95% CI, 2.00-128) in the youngest group. Among comorbidities and medications, alcoholism and taking benzodiazepines were also associated with dementia with elevated adjusted HRs of 3.05 (95% CI 2.17-4.28) and 1.23 (95% CI 1.14-1.32), respectively.

**Conclusions:**

Patients with CKD could have an elevated dementia risk. CKD patients with comorbidity deserve attention to prevent dementia.

## Background

Among all countries, the incidence of end-stage renal disease (ESRD) (41.5 per 100,000) has been the highest in Taiwan since 2000 [1-4]. There were more than 2 million ESRD patients worldwide in 2010 requiring dialysis or transplantation [5]. It is well known that patients with diabetes mellitus and/or chronic kidney disease are at high risk of developing ESRD [3-7]. The prevalence of CKD has been shown to be higher in older adults in Taiwan [2], United States [6], Japan [7] and internationally [8,9].

Dementia is a heterogeneous and multifactorial disorder, including vascular-type dementia and Alzheimer-type dementia [10]. The prevalence of dementia increases with age, from less than 1% in people aged 65 years to more than 25% in those aged 85 years or over [11,12]. Dementia has been a hidden health issue in Taiwan in the elderly population. The prevalence of the disorder in the elderly in Taiwan increased continuously over past decades, from 4.1% in 1980, to 6.1% in 1990, to 8.6% in 2000 and to 10.2% in 2007 [13]. It is therefore important to identify protective and risk factors for dementia to prevent this disease at an early stage. CKD has been proposed as a major factor of developing ESRD. It is also an independent risk factor for cognitive impairment but with conflicting findings among studies. Dementia and CKD have been recognized as comorbidities with higher incidence rate among the elderly [10], Sasaki et al. further found the incidence rate of dementia in Osaki area patients with CKD was much higher than the general Japanese population (24.0% vs. 9.0%) [14].

Recent studies in the US have also shown that impaired kidney function is associated with the increased prevalence of cognitive impairment [15,16]. Studies have identified age, hypertension and diabetes mellitus as independent factors in the association between kidney function and impaired cognition [14-17]. However, there were limited population based cohort studies on the association between CKD and the risk of dementia. To fill this research gap, we conducted this population based cohort study using claims data from the National Health Insurance (NHI) in Taiwan to explore issues of (i) the association between CKD and dementia in the aging population in Taiwan, (ii) the role of other comorbidities in the risk of dementia, (iii) the relationship between medications and the risk of dementia.

## Methods and materials

### Data sources

The National Health Insurance (NHI) of Taiwan is a universal health insurance program reformed in 1995, offers the coverage to 99% of the nation’s population of 23 million, and approximately 97% of hospitals and clinics have contracted with the NHI [18]. For research and administrative purposes, the National Health Research Institute, Taiwan, has used the claims data to establish various sets of database for public uses. This study used a longitudinal dataset including one-million insured people randomly selected from the whole population covered by the NHI. This dataset contains information on all medical services, such as ambulatory care claims, inpatient claims and prescription drugs and registry beneficiaries [18-20]. The privacy of all patients was protected by the encryption of personal identification. Using the unique scrambled personal identifications, we were able to link these to obtain medical histories and demographic variables. This study was exempted from full institutional review.

### Study subjects

In this study, we identified study subjects from 2000 to 2006 with newly diagnosed chronic kidney disease (CKD) as CKD cohort and those without any CKD diagnosis as non-CKD cohort. Subjects in the CKD cohort were identified using International Classification of Diseases 9^th^ Revision, Clinical Modification [ICD-9-CM], including 250.4(diabetes with renal manifestations), 274.1(gouty nephropathy), 403(hypertensive chronic kidney disease)- 404(hypertensive heart and chronic kidney disease), 440.1(disease of renal artery), 442.1(disease of renal artery), 447.3(hyperplasia of renal artery), 581(nephritic syndrome)-583(nephritis and nephropathy), 585(chronic renal failure)-588(disorders resulting from impaired renal function). Inpatients with at least one CKD diagnosis or outpatients with twice CKD service claims were included in the CKD cohort. Patients with codes of 283.1(hemolytic uremic syndrome), 572.4 (hepatorenal syndrome), 642.1(Hypertension secondary to renal disease, complicating pregnancy, childbirth, and the puerperium) and 646.2(Unspecified renal disease in pregnancy, without mention of hypertension) were excluded. Patients who had diagnoses of ESRD, renal transplantation therapy or dialysis therapy were also excluded. The first date of diagnosed CKD was the index date for the follow-up time measure. Subjects with dementia (ICD-9-CM 290.0, 290.1, 290.2, 290.3, 290.4, 294.1 and 331.0) prior to the index date or with missing information on gender or age were also excluded from this study.

For each CKD patient, two non-CKD subjects were randomly selected frequency matching with gender, age (every 5 years), index-year and index-month. The exclusion criteria used to select CKD cohort were used to select non-CKD cohort. We also avoid selecting subjects developing CKD in the non-CKD cohort, during the follow-up period. The follow-up time in person-years was calculated for each study subject until the occurrence of dementia, the end of 2009 or censored because of deaths or withdrawal from the insurance program. Other considered comorbidities at the baseline were hypertension (ICD-9-CM 401–405, A-code A260 and A269), diabetes mellitus (ICD-9-CM 250 and A-code A181), ischemic heart disease (ICD-9-CM 410–414, A-code A270 and A279), hyperlipidemia (ICD-9-CM 272.0, 272.1, 272.2, 272.3 and 272.4), tobacco use disorder (ICD-9-CM 305.1), alcoholism (ICD-9-CM 291, 291.2, 291.9, 303.9, 334.4, 980.0, V11.3, V79.1 and V61.41), morbid obesity (ICD-9-CM 278.0, 278.00, 278.01, 649.1, 783.1 and V77.8), atrial fibrillation (ICD-9-CM ), Parkinson's disease (ICD-9-CM 332 and A-code A221), cerebrovascular disease (ICD-9-CM 430–438, A-code A290-A294 and A299), major depressive disorder (ICD-9-CM 296.2 and 296.3), and elevated C-reactive protein screen (ICD-9-CM 790.95). The analysis of medication drugs related to dementia was performed for the use of benzodiazepine, aspirin, other NSAIDs and COX2.

### Statistical analysis

Data analysis first compared the demographic status and comorbidites between CKD and non-CKD cohorts. The Chi-square test was used to compare the difference in gender, age and baseline comorbidities between the CKD and non-CKD cohorts. Student’s *t*-test was used to compare the difference in mean age. Incidence rates of dementia for both cohorts, and the CKD cohort to non-CKD cohort incidence rate ratio (IRR) and 95% confidence interval (CI) were estimated by gender, age and follow-up time (< 2 and ≥ 2 years). We estimated incidence rates of dementia by comorbidity and medication, and used Cox proportional hazards regression model to estimate the specific hazard ratio (HR) and 95% confidence intervals of dementia for the CKD cohort compared to the non-CKD cohort. The Cox model was also used to estimate age, sex, comorbidity and medication specific crude HRs of dementia and the multivariate adjusted HRs. All analyses were performed using SAS software version 9.1 (SAS Institute Inc., Cary, NC), and the statistical significance level was set at two-sided *p* < 0.05.

## Results

There were more men than women in both cohorts (54.2% vs. 45.8%), with the mean age slightly greater in the CKD cohort than the non-CKD cohort (Table 1). Approximately 15% of subjects were young in 20–39 years of age. Subjects with CKD had a higher prevalence of all baseline comorbidities and medications (*p* < 0.0001). Table 2 shows that the incidence of dementia was higher in the CKD cohort than in the non-CKD cohort (9.30 vs. 5.55 per 1,000 person-years), with an IRR of 1.68 (95% CI 1.58-1.78). The incidence was slightly higher in women than in men, increased with age. But the age-specific relative risk was the highest for the youngest subcohort with an IRR of 16.0 (95% CI 2.00-128.0), and the lowest for the elderly with the IRR of 1.75 (95% CI 1.64-1.86). The IRR declined slightly after a 2-year follow-up.

**Table 1:** **Baseline characteristics between CKDcohort and non-CKD cohort**

	**non-CKD** **N = 74098**		**CKD** **N = 37049**		
	**n**	**%**	**n**	**%**	*P *value^*^
Sex					1.00
Women	33948	45.8	16974	45.8	
Men	40150	54.2	20075	54.2	
Age group (years)					1.00
20–39	10710	14.5	5355	14.5	
40–64	34742	46.9	17371	46.9	
≥65	28646	38.7	14323	38.7	
Mean (SD) (years)	57.5	(15.8)	58.3	(16.1)	<0.0001
Co-morbidities					
Hypertension	23702	32.0	21731	58.7	<0.0001*
Diabetes mellitus	6367	8.59	11517	31.1	<0.0001*
Ischemic heart disease	12896	17.4	12056	32.5	<0.0001*
Hyperlipidemia	7350	9.92	9762	26.4	<0.0001*
Tobacco use disorder	138	0.19	135	0.36	<0.0001*
Alcoholism	294	0.40	335	0.90	<0.0001*
Obesity	398	0.54	401	1.08	<0.0001*
Atrial fibrillation	638	0.86	740	2.00	<0.0001*
Parkinson's disease	790	1.07	680	1.84	<0.0001*
Cerebrovascular disease	8701	11.7	7876	21.3	<0.0001*
Major depressive disorder	852	1.15	652	1.76	<0.0001*
Elevated C-reactive protein	--		--		--
Medication					
Use of benzodiazepine	37902	51.2	25284	68.2	<0.0001*
Use of aspirin	6992	9.44	5822	15.7	<0.0001*
Use of other NSAIDs	63174	85.3	34698	93.7	<0.0001*
Use of COX2	14584	19.7	10901	29.4	<0.0001*

^*^Chi-square test comparing patients with and without CKD.

**Table 2:** **Incidence of dementia estimatedby sex, age, andfollow-up years for CKDand non-CKD cohorts**

	**Non-CKD**				**CKD**					
	**N**	**Case**	**Person-years**	**Incidence rate**^**†**^	**N**	**Case**	**Person-years**	**Incidence rate**^**†**^	**IRR**	**(95% CI)**
All	74098	2621	472301	5.55	37049	2013	216301	9.30	1.68	(1.58-1.78)
Sex										
Women	33948	1293	222306	5.82	16974	969	102586	9.45	1.62	(1.49-1.77)
Men	40150	1328	249995	5.31	20075	1044	113795	9.17	1.73	(1.59-1.87)
Age, years										
20–39	10710	1	72841	0.01	5355	8	36456	0.22	16.0	(2.00-128)
40–64	34742	262	234351	1.12	17371	298	111467	2.67	2.39	(2.03-2.82)
≥65	28646	2358	165109	14.3	14323	1707	68458	24.9	1.75	(1.64-1.86)
Follow-up time, year										
< 2	74098	696	144691	4.81	37049	651	68898	9.45	1.96	(1.77-2.18)
≥ 2	70600	1925	327610	5.88	32917	1362	147483	9.23	1.55	(1.43-1.67)

^†^ Incidence rate: per 1,000 person-years.

IRR (Incidence rate ratio): CKD vs. non-CKD (95% CI).

Table 3 presents the comorbidity-specific incidence rates of dementia in both cohorts and the CKD cohort to non-CKD cohort hazard ratios after controlling for age, sex, and medications. Most of comorbidities are associated with increased dementia incidence, the highest for CKD patients with the comorbidity of Parkinson's disease (50.9 per 1,000 person-years), followed by those with atrial fibrillation (29.9 per 1,000 person-years), cerebralvascular disease (25.1 per1,000 person-years), etc. These comorbidites were also associated with increased dementia incidence for non-CKD subjects.

**Table 3:** **Incidence of dementia estimatedby comorbidity for CKDand non-CKD cohorts**

	**Non-CKD**		**CKD**			
**Comorbidity**	**Case**	**Incidence rate**^**†**^	**Case**	**Incidence rate**^**†**^	**HR**	**(95% CI)**
Hypertension						
No	995	3.01	337	3.48	1.64	(1.45-1.86)
Yes	1626	11.5	1676	14.02	1.48	(1.38-1.58)
Diabetes mellitus						
No	2212	5.08	1203	7.83	1.52	(1.42-1.64)
Yes	409	11.2	810	12.9	1.42	(1.26-1.60)
Ischemic heart disease						
No	1680	4.23	921	6.05	1.63	(1.50-1.76)
Yes	941	12.5	1092	17.0	1.52	(1.39-1.65)
Hyperlipidemia						
No	2286	5.33	1491	9.36	1.65	(1.54-1.78)
Yes	335	7.78	522	9.14	1.43	(1.25-1.64)
Tobacco use disorder						
No	2620	5.55	2007	9.30	1.60	(1.51-1.70)
Yes	1	1.81	6	11.1	6.73	(0.81-56.1)
Alcoholism						
No	2608	5.54	1992	9.27	1.60	(1.50-1.69)
Yes	13	8.72	21	14.8	2.88	(1.41-5.91)
Obesity						
No	2614	5.56	2001	9.34	1.60	(1.51-1.70)
Yes	7	3.13	12	5.32	2.81	(1.04-7.58)
Atrial fibrillation						
No	2562	5.46	1929	9.03	1.59	(1.50-1.69)
Yes	59	19.2	84	29.9	1.67	(1.19-2.33)
Parkinson's disease						
No	2482	5.30	1876	8.78	1.61	(1.51-1.71)
Yes	139	38.0	137	50.9	1.30	(1.02-1.65)
Cerebrovascular disease						
No	1701	4.01	1048	5.89	1.59	(1.47-1.72)
Yea	920	19.2	965	25.1	1.41	(1.28-1.54)
Major depressive disorder						
No	2546	5.44	1949	9.15	1.61	(1.51-1.71)
Yes	75	16.5	64	18.8	1.44	(1.03-2.03)

^†^ Incidence rate: per 1,000 person-years.

HR: CKD vs. non-CKD (95% CI), adjsued for age, sex, use of benzodiazepine, aspirin, other NSAIDs and COX2.

The CKD cohort had a higher risk of dementia than the non-CKD cohort (adjusted HR 1.41, 95% CI 1.32-1.50) in the multivariate Cox model (Table 4). There was no significant different in the dementia risk between men and women. The adjusted hazard of dementia was much greater for the elderly (HR 133.0, 95% CI 68.9-256), compared with those in 20–39 years of age. CKD patients with the comorbidity of Parkinson's disease had a crude HR of 6.92 (95% CI 6.13-7.82), compared with the non-CKD cohort. The risk remained significant (HR = 1.80, 95% CI 1.59-2.04) in the multivariate analysis. However, the HR in the multivariable model was the highest for patients with alcoholism (HR = 3.05, 95% CI 2.17-4.28). Some other comorbidities could also predict the elevated hazard of dementia for patients with CKD, except for ischemic heart disease, hyperlipidemia, obesity, atrial fibrillation and the use of aspirin and other NSAIDs. The crude HR associated with hyperlipidemia was significant, but became protective in the multivariable analysis. Taking benzodiazepines were also associated with the dementia risk.

**Table 4:** **Cox model measured hazardratios and 95% confidenceintervals of dementia associatedwith CKD and covariates**

	**Crude**		**Adjusted**^**†**^	
**Variable**	**HR**	**(95%CI)**	**HR**	**(95%CI)**
Sex				
Women	1.00		1.00	
Men	0.94	(0.89-1.00)	0.97	(0.92-1.03)
Age, years				
20–39	1.00		1.00	
40–64	19.7	(10.2-38.0)	16.1	(8.35-31.2)
≥65	216	(112.-414)	133	(68.9-256)
CKD				
No	1.00		1.00	
Yes	1.68	(1.58-1.78)	1.41	(1.32-1.50)
Hypertension				
No	1.00		1.00	
Yes	4.10	(3.85-4.37)	1.17	(1.09-1.26)
Diabetes mellitus				
No	1.00		1.00	
Yes	2.14	(2.00-2.28)	1.25	(1.17-1.34)
Ischemic heart disease				
No	1.00		1.00	
Yes	3.11	(2.94-3.30)	1.00	(0.93-1.06)
Hyperlipidemia				
No	1.00		1.00	
Yes	1.35	(1.25-1.45)	0.89	(0.83-0.97)
Tobacco use disorder				
No	1.00		1.00	
Yes	1.00	(0.48-2.09)	--	
Alcoholism				
No	1.00		1.00	
Yes	1.78	(1.27-2.49)	3.05	(2.17-4.28)
Obesity				
No	1.00		1.00	
Yes	0.64	(0.41-1.00)	0.93	(0.59-1.46)
Atrial fibrillation				
No	1.00		1.00	
Yes	3.77	(3.19-4.45)	1.17	(0.99-1.39)
Parkinson's disease				
No	1.00		1.00	
Yes	6.92	(6.13-7.82)	1.80	(1.59-2.04)
Cerebrovascular disease				
No	1.00		1.00	
Yes	4.85	(4.57-5.14)	1.72	(1.61-1.84)
Major depressive disorder				
No	1.00		1.00	
Yes	2.68	(2.26-3.17)	1.95	(1.64-2.31)
Use of benzodiazepine				
No	1.00		1.00	
Yes	2.54	(2.37-2.71)	1.23	(1.14-1.32)
Use of aspirin				
No	1.00		1.00	
Yes	1.85	(1.71-2.00)	1.02	(0.95-1.11)
Use of other NSAIDs				
No	1.00		1.00	
Yes	1.54	(1.39-1.70)	1.01	(0.91-1.12)
Use of COX2				
No	1.00		1.00	
Yes	2.45	(2.31-2.60)	1.09	(1.02-1.16)

^†^ Adjusted for sex, age, CKD, hypertension, diabetes mellitus, ischemic heart disease, hyperlipidemia, alcoholism, obesity, atrial fibrillation, Parkinson's disease, cerebrovascular disease, major depressive disorder, use of benzodiazepine, aspirin, other NSAIDs and COX2.

## Discussion

This study revealed that the risk of dementia in the CKD cohort was higher than that in the non-CKD cohort yield a HR of 1.04 after controlling for demographic variables and medical conditions. This result is comparable with the findings in previous studies. Nickolas et al. found that the mild cognitive impairment in older adults with CKD is also a potentially modifiable risk factor for dementia [21]. Another survey revealed that moderate to severe CKD in stroke-free subjects was associated with white matter hyperintensity, which is resulted from inflammation and endothelial dysfunction and is a risk for dementia [22,23]. In addition, the present study supports previous findings of the relationship between impaired kidney function and cognitive decline [16,17,22,24-26]. Kahtri et al found that patients with kidney disease even with mild CKD 訂 e at an elevated risk of cognitive impairment [24].

It is important to note that a large proportion of CKD patients were young, aged 20-39 years, accounting for approximately 15% in the cohort. The young CKD patients to the young non- CKD patients IRR of dementia was near 9-fold greater than that for the elderly, reflecting CKD has a strong impact for young people. The corresponding IRR for 40-64 years was 13.6-fold greater than the elderly. Our data also showed that the risk of dementia declined with longer follow-up time, indicating dementia may develop in earlier period of CKD. Prevention care should be started earlier period including younger CKD patients.

This study also found several comorbidities and medications could predict dementia. The associations between blood pressure, diabetes and dementia and cognitive impairment have received much attention in studies, but the results are somewhat in conflict [25-32]. High blood pressure increases the risk of dementia in the elderly [26,30]. Xu et al. found that the risk of dementia was especially high for patients with diabetes mellitus and severe systolic hypertension [30]. Our results echo most of previous findings that hypertension and diabetes are independent predictors of dementia [32].

An earlier study has found that very old women with myocardial infarction are 5-time more likely prone to dementia than those without the disease [33]. The cross-sectional analysis from the Rotterdam study showed that the prevalence of dementia was increased with the degree of peripheral arterial atherosclerotic disease. The odds ratio for cognitive decline in those with severe atherosclerosis was 3:0 [34]. Our study showed that CKD patients with ischemic heart disease were 2.8-fold (17.0 vs. 6.05 per 1000 person-years) more likely than CKD patients without the comorbidity for the risk of dementia. This association became insignificant in the multivariate analysis.

Among comorbidities, alcoholism has the strongest association with dementia in this study. Breteler found a significant relationship between alcohol intake and the risk of vascular disease [35]. Those with moderate alcohol intake may not at an increased risk of dementia [36-38]. The congnitive impairment due to alcohol addition has been 臼 ported as alcohol dementia [29,39]. Alcoholism was less prevalent in our study population,but it is an important risk factor of dementia.

In addition to alcoholism, hypertension and diabetes, we also found that cerebrovascular disease, Parkinson's disease and major depressive disorder were significant comorbidities associated with dementia. Studies have reported cerebrovascular disease as a major contributor to later-life dementia [31,40-44]. Our findings reiterate previous pathological investigations. The later epidemiological studies may have raised the possibility that cerebrovascular disease and dementia are two disorders causally related [42]. Parkinson's disease and the major depressive disorder are relatively less important than cerebrovascular disease in the association with the dementia risk [45-47].

Hyperlipidemia had a significant risk of dementia in the univariate model but it became a protective effect after being adjusted. In subjects without hyperlipidemia, the incidence of dementia in CKD patients is higher than non-CKD patients (9.36 vs. 5.33 per 1,000 person-years). However ,the incidence in CKD patients with hyperlipidemia declined to 9.14 per 1,000 person-years. The association between hyperlipidemia and dementia is not clear. The colinearity of hypertension and hyperlipidemia may explain the phenomenon.

The significant relation between dementia and medications of benzodiazepines agents and aspirin is another important finding in this study. Previous studies have reported that the long-term use of benzodiazepines agents is responsible for moderate-to-large weighted risk of dementia [48-52]. The aspirin use might pose an increased risk of intracerebral hemorrhages [53]. It is not clear whether this is the mechanism increases the risk of dementia.

### Limitations

The insurance claims data provided no measures of serum creatinine and microalbuminuria, and we were unable to evaluate the dementia risk based on kidney function. CKD patients at an earlier stage might not be included in the study. This may, therefore, have resulted in estimating the risk for patients with more severe condition of CKD. Second, as diagnosis of CKD and dementia requires long-term observation for clinical manifestation, the seven-year or less observation period in this study may be insufficient to estimate the dementia risk with the full course of natural history. The associated risk measures may be thus underestimated.

Third, this study included conmorbidity information at the baseline, prior to the date of establishing the study cohorts. Comorbidities developed during the follow-up period may vary and the estimation of dementia risk may vary.

## Conclusions

This study evaluated the risk of dementia in associations with CKD and several medical conditions and medications for the Asian population, using representative population-based data. We have demonstrated that patients with CKD are at a significantly higher risk of dementia than those without CKD. Other comorbidity may also interact with CKD and increase further the risk of dementia. CKD is a much more prevalent disorder in the current study population than populations of many other countries. Studies are needed for patients with CKD not only for the prevention of developing end stage renal disease, but also for preventing the progression of dementia. The identification of comorbidities and medications may yield important prevention opportunity for reducing dementia development. Our data suggest that intervention should be applied to young patients as well.

## Competing interests

The authors declare that they have no competing interests.

## Authors’ contributions

Kao-Chi Cheng: (1) substantial contributions to the conception of this article; (2) planned and conducted the study; (3) initiated the draft of the article and critically revised it; Yu-Lung Chen: (1) substantial contributions to the conception of this article; (2) planned and conducted the study; (3) initiated the draft of the article and critically revised it;Shih-Wei Lai: (1) participated in data interpretation; (2) critically revised the article;Chih-Hsin Mou: (1) conducted data analyses; (2) data interpretation Pang-Yao Tsai: (1) conducted data analyses; (2) data interpretation; Fung-Chang Sung: (1) obtained study materials with substantial contributions to the study concept and design; (2) conducted data analysis and data interpretation; (3) critically revised the article and response to the Reviewers’ comments. All authors read and approved the final manuscript.

## Pre-publication history

The pre-publication history for this paper can be accessed here:

http://www.biomedcentral.com/1471-2369/13/129/prepub
